# Triple Stiffness: A Bioinspired Strategy to Combine Load‐Bearing, Durability, and Impact‐Resistance

**DOI:** 10.1002/advs.202004338

**Published:** 2021-03-18

**Authors:** Ali Khaheshi, Stanislav Gorb, Hamed Rajabi

**Affiliations:** ^1^ Functional Morphology and Biomechanics Institute of Zoology Kiel University Kiel 24118 Germany

**Keywords:** adaptive systems, buckling, deformability regimes, tuneable stiffness, variable rigidity

## Abstract

Structures with variable stiffness have received increasing attention in the fields of robotics, aerospace, structural, and biomedical engineering. This is because they not only adapt to applied loads, but can also combine mutually exclusive properties. Here inspired by insect wings, the concept of “triple stiffness” is introduced and applied to engineering systems that exhibit three distinct deformability regimes. By implementing “flexible joints,” “mechanical stoppers,” and “buckling zones,” structures are engineered to be not only load‐bearing and durable, but also impact‐resistant. To practice the performance of the design concept in real‐life applications, the developed structures are integrated into 3D printed airplane wing models that withstood collisions without failure. The concept developed here opens new avenues for the development of structural elements that are load‐bearing, durable, and impact‐resistant at the same time.

## Graphical abstract

Inspired by insect wings, the concept of ‘triple stiffness’ is introduced and implemented into structures that reach a compromise between load‐bearing, durability, and impact‐resistance. The structures are further incorporated into the wings of 3D printed airplane models that can withstand collisions and quickly recover. This study inspires the design of novel adaptive structures in robotics, aerospace, biomedical, and structural engineering.

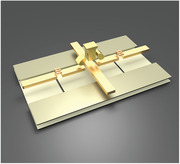

## 1 Introduction

Flexibility and stiffness are two key characteristics of a structural element. However, they are often mutually exclusive; changing one of them causes a reciprocal change in the other one. A solution to combine the flexibility and stiffness in a mechanical system lies in an adaptive trade‐off between them. Interestingly, such a combination can often be found in biological structures.^[^ ^1^, ^2^, ^3^ ^]^ Insect wings, for instance, represent a remarkable example.^[^ ^4^, ^5^ ^]^ They constantly respond to the applied loads, by adjusting their stiffness and flexibility, through different design strategies, which often include structural gradients,^[^ ^6^ ^]^ flexible joints,^[^ ^7^ ^]^ and mechanical interlocking.^[^ ^8^, ^9^ ^]^

In recent years, researchers have applied either of the above‐mentioned strategies to engineering structures.^[^ ^10^, ^11^ ^]^ This has usually ended up with mechanical systems that display two deformability regimes (i.e., stiffness levels), depending on the magnitude of applied loads. The variable, yet reversible, stiffness levels have provided such “double‐stiffness” structures with a major advantage over the conventional single stiffness structures: they can adapt to applied loads. That is why the “double‐stiffness” structures have found plenty of applications in biomedical, aerospace, robotics, structural engineering, etc.^[^ ^12^ ^]^

“Double‐stiffness” structures present multiple advantages over commercially available components that gradually change stiffness in response to loads (e.g., nonlinear springs). A piece‐wise linear stiffness change, as seen in many “double‐stiffness” structures, makes them particularly attractive for practical applications. This is due to three main reasons. First, there is no time delay in the process of stiffness change of “double‐stiffness” structures, which enables the system to be highly dynamic.^[^ ^12^, ^13^ ^]^ Second, the linear response of “double‐stiffness” structures makes them more predictable and therefore, facilitates their modeling and controllability for robotic applications.^[^ ^14^ ^]^ Third, the properties of “double‐stiffness” structures in each deformability regime can be readily tuned by adjusting their geometric features. This enables them to reach a wide range of variable stiffness, which is especially important for shape morphing systems.^[^ ^15^ ^]^

The approaches to achieve a reversible change in the stiffness, however, could be highly different. One scenario is that an initially stiff, that is, load‐bearing, structure turns into a flexible one to prevent failure under excessive loading. This transition could be triggered, for instance, by an external force upon a mechanical impact.^[^ ^16^ ^]^ In another scenario, a flexible structure, turns into a stiff one to restrict deformability. Khaheshi et al.,^[^ ^11^ ^]^ for example, developed a durable structure that is initially flexible, but becomes stiff through an interlocking effect to prevent detrimental large deformations. Each of the two approaches, however, represents a drawback. The impact‐resistant structures developed based on the former approach are often less durable than those implementing the latter approach, when subjected to the same displacement. In contrast, the durable structures developed based on the latter approach could fail under mechanical impacts or excessive stresses. How can an engineering structure gain the advantages of the two described approaches, that is, load‐bearing, durability, and impact‐resistance?

**Figure 1:**
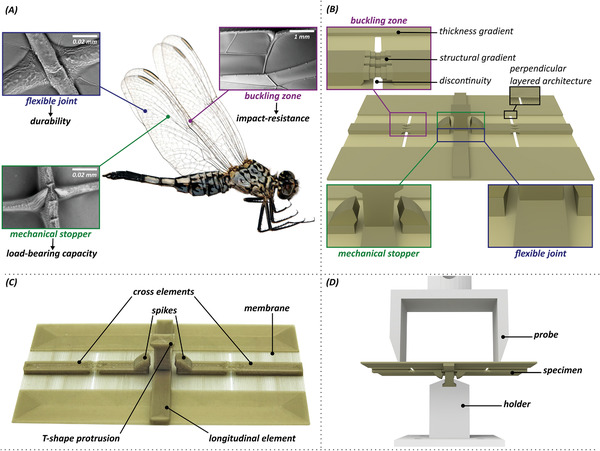
Inspiration, design, fabrication and mechanical testing of “triple stiffness” structures. A) Design strategies of a dragonfly (*Acisoma panorpoides*, Reproduced with permission.^[^ ^17^ ^]^ Copyright 2018, Elsevier) wing: flexible joint, mechanical stopper (i.e., spike), and buckling zone (i.e., costal break or nodus). B) Wing‐inspired design strategies included in the “reference model.” C) 3D printed prototype of the “reference model” with the indication of the key structural elements. D) Experimental setup used in the static, dynamic, and fatigue tests.

This question prompted the current study. To find the answer, we used bioinspired strategies (**Figure** **1**A), to engineer a structure that had three stiffness levels. The developed structure exhibited a low stiffness under small loads, became stiff under higher loads, and turned again into a low stiffness regime upon impacts/excessive loadings to prevent mechanical failure. When the load was removed, the structure bounced back to its original form through a reversible transition (Video S1, Supporting Information).

## 2 Results

### 2.1 Modeling and Fabrication

We used computer aided design (3D computer graphics software Blender v. 2.8) and 3D printing (Prusa i3 MK2.5S) to design and fabricate our structure. The developed 3D model and its 3D printed prototype are illustrated in Figure 1B,C. As seen, we implemented three bioinspired strategies into our design. These included flexible joints, to allow the deformability under relatively small loads, spike‐shaped mechanical stoppers, to interlock and increase the stiffness under higher loads, and buckling zones, to enable the structure to reversibly buckle after mechanical impacts (Figure 1A,B).

Our main model, called as the “reference model” was composed of a membrane and three reinforcing elements, which crossed each other and created a joint‐like structure at the center of the model (Figure 1B,C). There were two spikes at one end of the two cross elements, which provided an interlocking effect in contact with a T‐shaped protrusion in the middle of the longitudinal element. A buckling zone was developed by designing a structural gradient, in form of dog bone‐shaped elements, in the middle of the both cross elements, where a discontinuity was also included in the membrane (Figure 1B). A thickness gradient was also introduced to the lateral edges of the membrane, as another component of the buckling mechanism. An exploded view of the model is presented in Figure S1, Supporting Information. A concentric filling pattern was used while 3D printing the model, which resulted in a perpendicular layered architecture. We used PLA/PHA filaments (filament diameter: 1.75 mm, nozzle temperature: 195–220 °C, heatbed temperature: 50–60 °C, 3D print speed: 40–100 mm s^−1^, Colorfabb) for 3D printing of the model (nozzle diameter/extrusion width: 0.4 mm, layer height: 0.2 mm, printing time: ≈20 min, dimensions: 8 × 5 × 1.1 cm^3^, weight: 3.8 g).

Three other models were developed by the removal of one of the design strategies from the “reference model”: i) “no flexible joint model,” by the removal of the flexible joints between the reinforcing elements; ii) “no mechanical stopper model,” by the removal of the spikes and iii) “no buckling model” by the removal of the buckling zones (i.e., structural gradient, thickness gradient, and discontinuity) (Figure S2, Supporting Information). The three models were used to evaluate the influence of the removal of each of the mentioned design strategies on the mechanical performance of the “reference model” (Video S2, Supporting Information).

### 2.2 Mechanical Performance of the Developed Structures under Different Loading Conditions

In order to characterize the mechanical behavior of our 3D printed structures under loading, we performed three sets of experiments: i) static, ii) dynamic and iii) fatigue tests to measure their load‐bearing capacity, impact‐resistance, and durability, respectively (Video S2, Supporting Information). For the static and dynamic tests, the models were fixed at the T‐shape protrusion in a mechanical holder (Figure 1D). A displacement of 13 mm was applied to the both cross veins using a customized two‐tip probe connected to a 500 N load cell (Xforce HP load cell, Zwick Roell) of a mechanical testing machine (Zwick Roell). This allowed us to fully capture the different deformability regimes of the models. The loading velocity was 1 mm s^−1^ in the static tests, to ensure quasi‐static loading, and 30 mm s^−1^ in the dynamic tests, as it was the maximum velocity at which the machine could reliably operate. The unloading velocity was set at 1 mm s^−1^ for both types of tests. This velocity was found to be slow enough to minimize effects of viscoelasticity. The same setup was used in fatigue tests. The specimens were subjected to 6.5 mm displacement (equal to half of the displacement applied in the static and dynamic tests). The test continued until a stabilized response was reached. For all tested specimens this happened below 100 loading cycles (i.e., low‐cycle fatigue). The force in the last loading cycle was demonstrated as a percentage of the force in the first loading cycle, and used as a measure of the durability (i.e., preserved load‐bearing) of the specimens. In each set of experiments, at least three specimens of each type were tested.

### 2.3 Comparative Analysis of the Developed Structures

**Figure 2:**
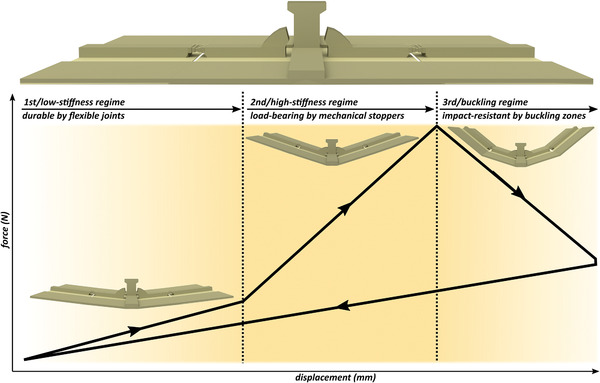
Schematic force‐displacement graph of the “reference model.” The plot shows three different deformability regimes due to the inclusion of three design strategies of the flexible joints, mechanical stoppers, and buckling zones. The initial state of the “reference model” before loading is demonstrated above the graph.

As seen in the schematic force–displacement curve and the one obtained from the static tests, our “reference model,” with the features shown in Figure 1B,C, exhibits three deformability regimes (**Figure** **2**). The deformation of the model under loading starts by the rotation of the cross elements about their joints with the longitudinal element. Due to the flexibility of these joints, the model freely deforms and exhibits a low stiffness of 0.70 ± 0.05 N mm^−1^ (high‐deformability/low‐stiffness regime in Figure 2). As the loading increases, the spikes on the cross elements move closer to the T‐shape protrusion until they contact. The mechanical contact between the spikes and the protrusion results in an interlocking effect, which prevents further deformation at the joints. As a result, the stiffness of the model increases and the model enters into a load‐bearing phase (low‐deformability/high‐stiffness regime in Figure 2). In this regime, the stiffness and, consequently, the load‐bearing capacity of the structure are drastically increased by ≈3.5 times (2.35 ± 0.13 N mm^−1^). When the loading exceeds a set threshold (here ≈12 N), at which the maximum load‐bearing is reached, the cross elements suddenly buckle and the model flexes to prevent failure (buckling regime in Figure 2). Upon the load removal, the structure returns to its original form without failure.

**Figure 3:**
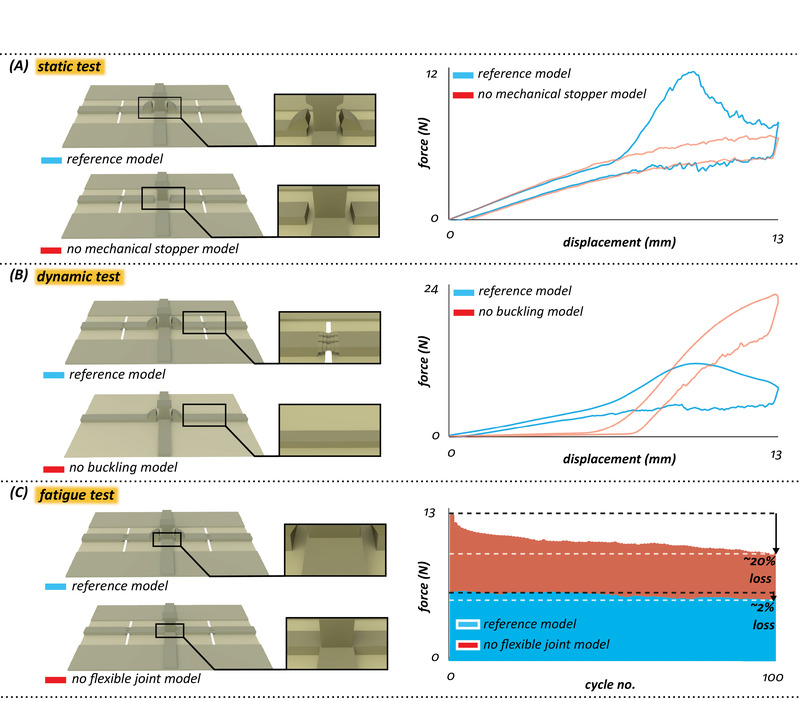
Mechanical behavior of the “reference model” versus “no mechanical stopper model,” “no buckling model,” and “no flexible joint model.” A) Force–displacement curves obtained from static tests for the “reference model” and “no mechanical stopper model.” B) Force–displacement curves obtained from dynamic tests for the “reference model” and “no buckling model.” C) Force‐cycle diagrams obtained from fatigue tests for the “reference model” and “no flexible joint model.”. The illustrated graphs are the average data of three specimens from each experiment.

We compared the behavior of the “reference model” under the static loading with that of the “no mechanical stopper model” (**Figure** **3**A). The removal of the spikes in the “no mechanical stopper model” resulted in a structure with only one deformability regime that has a stiffness of 0.60 ± 0.03 N mm^−1^. This is almost equal to the stiffness of the “reference model” in the first deformability regime and one‐fourth of that of the “reference model” in the high‐stiffness regime. With that said, the “no mechanical stopper model” lost its load‐bearing capacity in comparison with the “reference model.”

The “reference model” showed a similar response to the dynamic loading, as that observed in the static tests. The model again went through the three deformability regimes described earlier (Figure 3B). To quantify the performance of the model under dynamic loading, we measured its residual displacement, which was 0.68 ± 0.13 mm. We then compared the behavior of the “reference model” with that of the “no buckling model” (Figure 3B). The absence of the buckling zones in the “no buckling model” resulted in a structure that had two deformability regimes. The lack of the third deformability regime, that is, buckling regime, led to a residual displacement of 2.43 ± 0.15 mm, which is more than three times higher than that measured in the “reference model.” This indicated that the “no buckling model” was no longer impact‐resistant. We should also mention that the residual displacement measured here could be caused by a combination of both plastic and viscoelastic deformations. However, we did not find any sign of plasticity in the “reference model” after loading, whereas the “no buckling model” underwent obvious plastic deformation. Nevertheless, we would like to emphasize that the term “reversible buckling” in the manuscript is used in a comparative sense.

The results also show a difference in the behavior of the two models in the first deformability regime, which is due to the absence of the thickness gradient in the “no buckling model.” The removal of this thickness gradient, which is a component of the buckling mechanism, has made the “no buckling model” less load‐bearing than the “reference model” in the first regime.

The force‐cycle diagrams of the “reference model” obtained from the fatigue tests are shown in Figure 3C. At the end of the experiments, the “reference model” maintained 98.27 ± 1% of its load‐bearing capacity. To estimate the effect of the flexible joint on the durability of the “reference model,” we compared the results with those of the “no flexible joint model” (Figure 3C). We could see here that the removal of the flexible joint, that is, the first deformability regime, reduced the durability of the “no flexible joint model.” The “no flexible joint model” maintained only 79.67 ± 2.9% of its original load‐bearing capacity. We should mention that here both models were subjected to a displacement that did not result in activation of the spikes in the “reference model.” This was necessary to demonstrate the influence of the flexible joints, which were intended to enhance the durability of the structure, independent from the mechanical role of the spikes.

### 2.4 The Bioinspired Solution in Application

**Figure 4:**
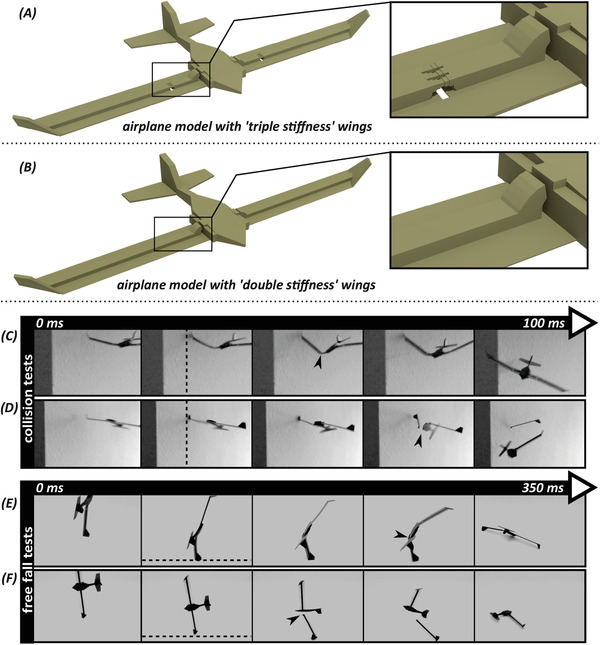
Design of the airplane models and their performance in impact tests. A) A perspective view of the airplane model with “triple stiffness” wings. B) A perspective view of airplane model with “double stiffness” wings. C,D) Collision tests. Snapshots from high‐speed video recordings of collision tests on the airplane model with C) “triple stiffness” wings and on the airplane model with D) “double stiffness” wings. E,F) Free fall tests. Snapshots from high‐speed video recordings of free fall tests on the airplane model with E) “triple stiffness” wings and on the airplane model with F) “double stiffness” wings. The arrowheads indicate the occurrence of the buckling and fracture in the airplane models with “triple stiffness” and “double stiffness” wings, respectively. The dashed lines show the rigid obstacle (i.e., wall) and the ground in the collision and free fall tests, respectively.

In order to practice the application of the “reference model” in real‐life, we designed and fabricated an airplane model. We implemented the “reference model” into the wings of our airplane (**Figure** 4A and Figure S3, Supporting Information). The design features of the “reference model” were slightly modified to enable wings to resist flight forces, but buckle upon excessive loads. As a comparison, we also fabricated an airplane in which “no buckling model” was replaced with the “reference model.” We referred to this type as airplanes with “double stiffness” wings (Figure 4B and Figure S4, Supporting Information). We carried out two types of experiments: i) collision tests by crashing the airplanes with a rigid obstacle and ii) free fall tests by dropping them from a constant height of 50 cm (Figure 4C–F and Video S3, Supporting Information). All experiments were recorded with a digital camera (SONY RX10 iii) at 500–1000 fps. The results showed that the airplanes with the “double stiffness” wings broke upon impact in both the collision and free fall tests, while the airplanes with the “triple stiffness” wings survived the impact in both of the tests through a reversible buckling (Figure 4C–F and Video S3, Supporting Information).

## 3 Discussion

By being stiff, on one hand, a structure can carry operational loads. By being flexible, on the other hand, it can deform upon external stimulus. Up to now, conventional structures have often been designed to satisfy either of the above‐mentioned functions. Since a certain frame of tasks is usually predefined for structures to fulfill, they are not able to adapt to a change in their tasks. In recent years, however, we have learned that by applying Nature‐inspired solutions we may be able to make a trade‐off between the stiffness and flexibility in engineering systems.^[^ ^2^, ^18^, ^19^ ^]^ The solution lies in adaptive structures that can tune their properties, particularly stiffness, in response to applied stresses. Such adaptive structures can perform multiple tasks and, therefore, are multifunctional. That is why they are receiving increasing attention in the fields of robotics, aerospace, structural engineering, etc.^[^ ^20^, ^21^, ^22^ ^]^

Due to the high potential of adaptive structures, there have been many efforts in recent years to develop structures with variable stiffness. Previous studies have used different strategies, such as layer jamming,^[^ ^23^, ^24^ ^]^ kinematic interaction of multiple surfaces,^[^ ^10^ ^]^ pre‐stretched sandwiched layers of multiple materials,^[^ ^16^ ^]^ flexible matrix composites filled with pressurizing fluid,^[^ ^25^ ^]^ and bistable mechanisms with two stable equilibrium states.^[^ ^22^, ^26^, ^27^, ^28^, ^29^ ^]^ In contrast to these elaborate, but complex, approaches, our method based on 3D printing of a single material is a rather simpler, faster, and less costly way to fabricate a structure with variable stiffness levels.

In our “reference model,” three design strategies were used: (i) flexible joints, (ii) mechanical stoppers, and (iii) buckling zones. Our results confirmed that the implementation of flexible joints enhanced the durability of the 3D printed models, in comparison with the “no flexible joint model.” This is because the flexible joints reduce the stress concentrations at the junctions of reinforcing elements. This finding is supported by a previous report, which showed that the stress within flexible joints of insect wings is up to 80 times smaller than that developed in fused joints under the same loading condition.^[^ ^30^ ^]^ Thus, flexible joints reduce the risk of material failure, either in the form of plasticity or fracture.^[^ ^11^ ^]^ However, a flexible structural element, although being durable, cannot carry loads. Hence, the inclusion of mechanical stoppers helped to limit the flexibility of the “reference model” and enhance its load‐bearing capacity. Despite being less load‐bearing than fully rigid structures or less durable than fully flexible ones, our design represents a novel bioinspired concept, which makes a compromise between the two properties that are mutually exclusive. A key benefit of our design is that the mechanical stoppers can be geometrically adjusted, in order to fine‐tune the timing and magnitude of stiffness change.^[^ ^11^ ^]^

Insect wings, particularly dragonfly wings, inspired our design strategy, that is, combination of the flexible joints and mechanical stoppers. As mentioned earlier, insect wings represent remarkable examples of a balance between load‐bearing and durability. This balance is achieved through a compromise between stiffness and flexibility; Wings need to be stiff enough to withstand aerodynamic loads and flexible enough to deform without failure.^[^ ^4^, ^5^, ^31^, ^32^ ^]^ Joint‐like structures play a key role in this regard. Due to their “weaker” structure or material, the joints are often more flexible than other wing components.^[^ ^7^, ^33^, ^34^ ^]^ However, joint‐associated spikes, which resemble our mechanical stoppers, provide the joints with the stiffness necessary to withstand aerodynamic forces.^[^ ^9^, ^30^, ^32^ ^]^ The same principle is valid for most engineering structural elements. This is the main reason why engineering structures are not made of the strongest materials; strong materials cannot deform readily and, therefore, tend to be brittle.^[^ ^2^, ^35^ ^]^ A similar design strategy as that used in insect wings, that is, joint‐like structures at the junctions of reinforcing elements, could help to improve the resilience of brittle composites and their resistance against fracture.

To enhance the resistance of our developed structures against mechanical impacts, we implemented a buckling mechanism into our design. This was done by the inclusion of a discontinuity and two structural gradients, which provided the “reference model” with the instability needed under mechanical impacts. While the discontinuity and the structural gradient implemented in the cross elements facilitated the occurrence of the buckling, a thickness gradient, in the form of two marginal strips at the edges of the membrane (Figure 1B), helped the structure to quickly return to the original state after mechanical impacts. The initiation of the buckling could be further adjusted by strengthening or weakening the structural gradient in the buckling zone. A perpendicular layered architecture with a concentric filling pattern helped to both facilitate the buckling mechanism and reduce the risk of crack propagation within the membrane. We should mention that the design strategies that enabled our models to buckle under excessive loadings are not completely similar to those of insect wings, which additionally include unsupported veins, shallow pleats, and graded properties.^[^ ^36^ ^]^

We implemented our “triple stiffness” structure, that is, the “reference model,” into the wings of airplane models and subjected them to mechanical impacts (Video S3, Supporting Information). The results indicated the significance of our design strategies to prevent catastrophic failures in a real‐life application. We observed that implementing a buckling zone in the wings of the airplane models helped them to survive crashes and quickly recover from them. Although one might argue that the failure of the airplane with “double stiffness” wings is due to the presence of the mechanical stoppers (i.e., spikes), without them the wings would not be load‐bearing enough. Replacing the flexible joint with a fused joint, without substantial increase of the material, would still result in wing failure, in form of plasticity, after a collision.

Here we did not analyze the dynamics of the 3D printed airplanes from the aspect of stability, since our main aim was to examine their resistance to mechanical impacts. However, we would like to build on our own previous work,^[^ ^11^ ^]^ in which we showed that combining the flexible joints and mechanical stoppers enhanced the flight stability of 3D printed “double‐stiffness” kites under wind gusts. This is mostly because, by slight changes in the configuration, the kites could automatically adapt to the changing flight conditions. We expect a similar effect to occur here. Although, taking into account the inherent difference between the free flight of airplanes and tethered flight of kites, this conclusion should be interpreted with caution.

Scalable manufacturing of adaptive structures with variable stiffness levels and their integration with strictly rigid conventional systems still present considerable technical challenges.^[^ ^37^, ^38^ ^]^ Some of these challenges are associated with the manufacturing processes, control strategies, long‐term durability, and efficiency of the mechanical parts when applied at different scales.^[^ ^19^, ^21^, ^38^ ^]^ The excellent mechanical performance of our “triple stiffness” structure along with its low‐cost merits and the ability to tune properties within a single material system are characteristics that make it a suitable candidate for scalable development. In addition to these, the deformations exhibited by our structure are fully passive and, hence, there is no requirement for a complex control strategy. The use of manufacturing techniques other than 3D printing by fused deposition modeling, as that used here, and the application of advanced structural composites, such as carbon fiber reinforced polymers, can further improve the performance of our bioinspired design. This is because of the quality issues associated with low‐cost 3D printed parts, which often contain small defects and coarse grains that negatively influence their specific stiffness.^[^ ^39^ ^]^ Hence, in summary, we expect that our design can be mass produced and implemented to high‐performance adaptive systems.

In the end, we conclude that our design strategy is an efficient, yet convenient, solution for the development of engineering structures that combine load‐bearing, durability, and impact‐resistance. These are the three key features of any structural element in order to properly function and survive “extreme” conditions. As a promising outlook, the implementation of the “triple stiffness” structures in wings of flapping robots or drones could facilitate their widespread use in civilian applications, such as safety inspection, environmental preservations, etc.^[^ ^40^ ^]^ The use of variable stiffness structures could also benefit the performance of conventional fixed‐wings aircrafts at different flight conditions.^[^ ^41^ ^]^ The concept developed here could inspire the design of novel adaptive structures in robotics, aerospace, biomedical and structural engineering.

Video S4, Supporting Information summarizes our study, including the underlying motivations, methodology, obtained results, and its contribution to the field.

## Conflict of Interest

The authors declare no conflict of interest.

## Author Contributions

Conceptualization: A.K., S.N.G., H.R.; Supervision: S.N.G., H.R.; Investigation: A.K., H.R.; Data Curation: A.K., H.R.; Formal Analysis: A.K.; Funding Acquisition: A.K., S.N.G., H.R.; Methodology: A.K., H.R.; Project Administration: H.R.; Resources: S.N.G.; Validation: A.K.; Visualization: A.K., H.R.; Writing—Original Draft Preparation: A.K.; Writing–Review & Editing: A.K., S.N.G., H.R.

## Supporting information



Supporting Information

Supplemental Video 1

Supplemental Video 2

Supplemental Video 3

Supplemental Video 4

## Data Availability

All supporting data are available in the article and on: https://doi.org/10.6084/m9.figshare.13013549. The data that support the findings of this study are available from the corresponding author upon reasonable request.
