# Supplementary material for: Triple Stiffness: A Bioinspired Strategy to Combine Load‐Bearing, Durability, and Impact‐Resistance
Source: Adv Sci (Weinh). 2021 Mar 18;8(11):2004338. doi: 10.1002/advs.202004338 (PMC8188221; doi:10.1002/advs.202004338)
Supplement: Supplementary file 1 — Supporting Information [file ADVS-8-2004338-s001.pdf]

© 2021 Wiley-VCH GmbH

ADVANCED SCIENCE
Open Access

Supporting Information

for *Adv. Sci.*, DOI: 10.1002/advs.202004338

Triple stiffness: A bioinspired strategy to combine load-bearing, durability and impact-resistance

Ali Khaheshi, Stanislav Gorb, Hamed Rajabi*

Supplementary materials

Fig. S1. An exploded view of the 'reference model' to demonstrate the relationship between its key components.

Fig. S2. The developed 3D printed models: (A) 'reference model', (B) 'no mechanical stopper model', (C) 'no buckling model', (D) 'no flexible joint model'.

Fig. S3. 3D printed airplane model with 'triple stiffness' wings.

Fig. S4. 3D printed airplane model with 'double stiffness' wings.

Vid. S1. A visual representation of the mechanical performance of triple stiffness structure (i.e. 'reference model') under loading.

Vid. S2. Mechanical performance of the 'reference model' vs. 'no mechanical stopper model', 'no buckling model' and 'no flexible joint model' during static, dynamic and fatigue tests, respectively.

Vid. S3. Collision and free fall tests of airplane models with 'triple stiffness' and 'double stiffness' wings.

Vid. S4. A summary of the article for the general audience.

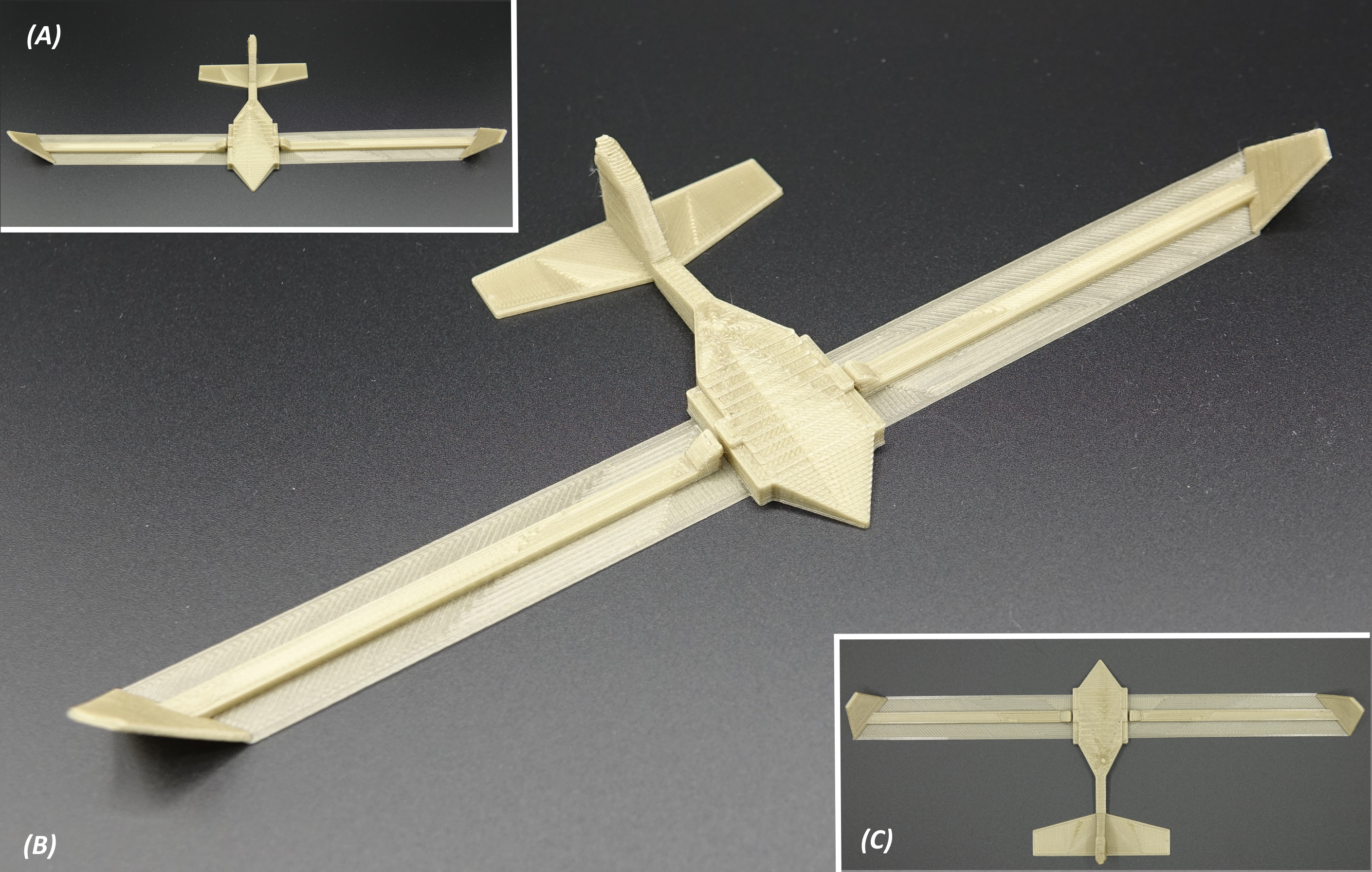
(A)
(B)
(C)

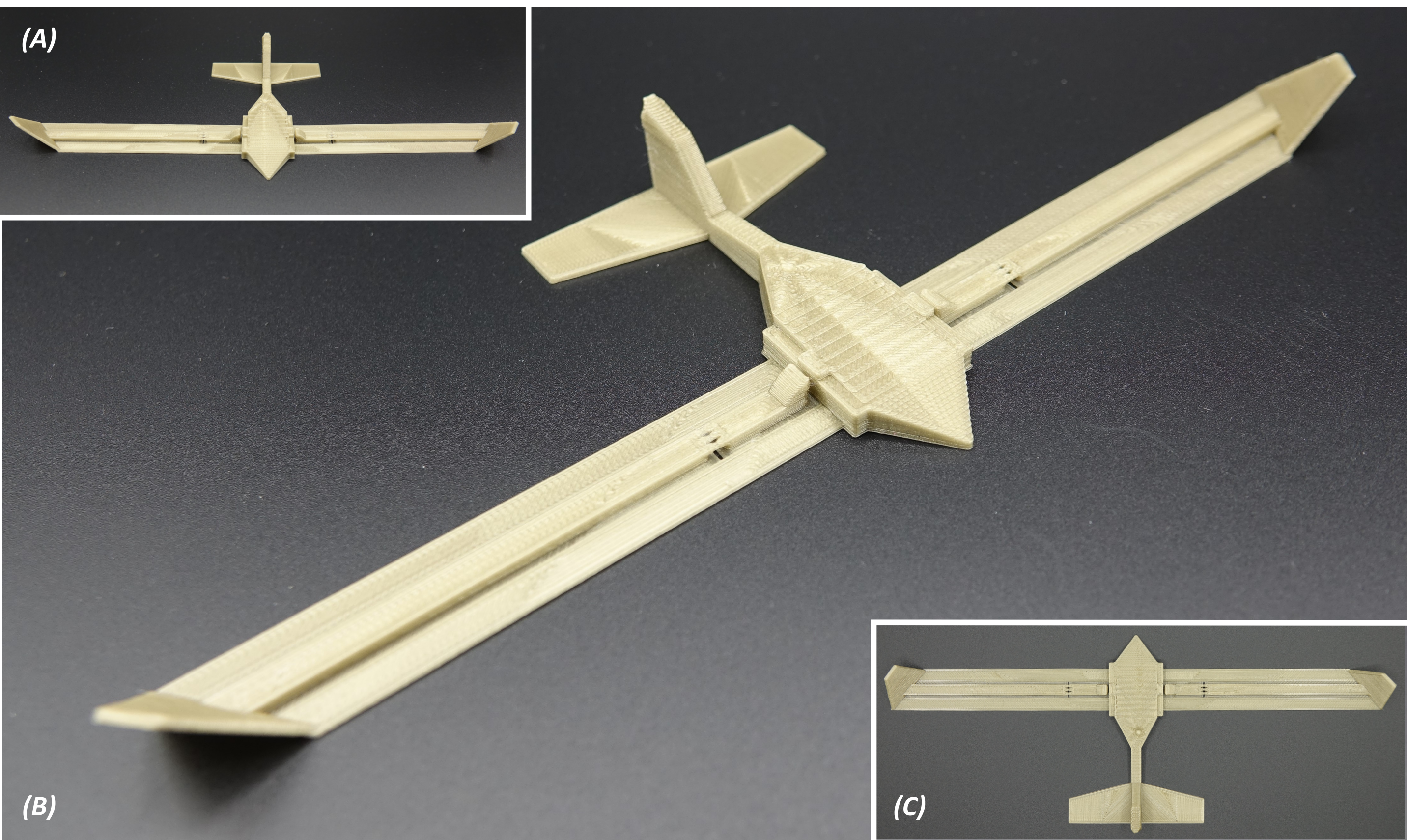
(A)
(B)
(C)

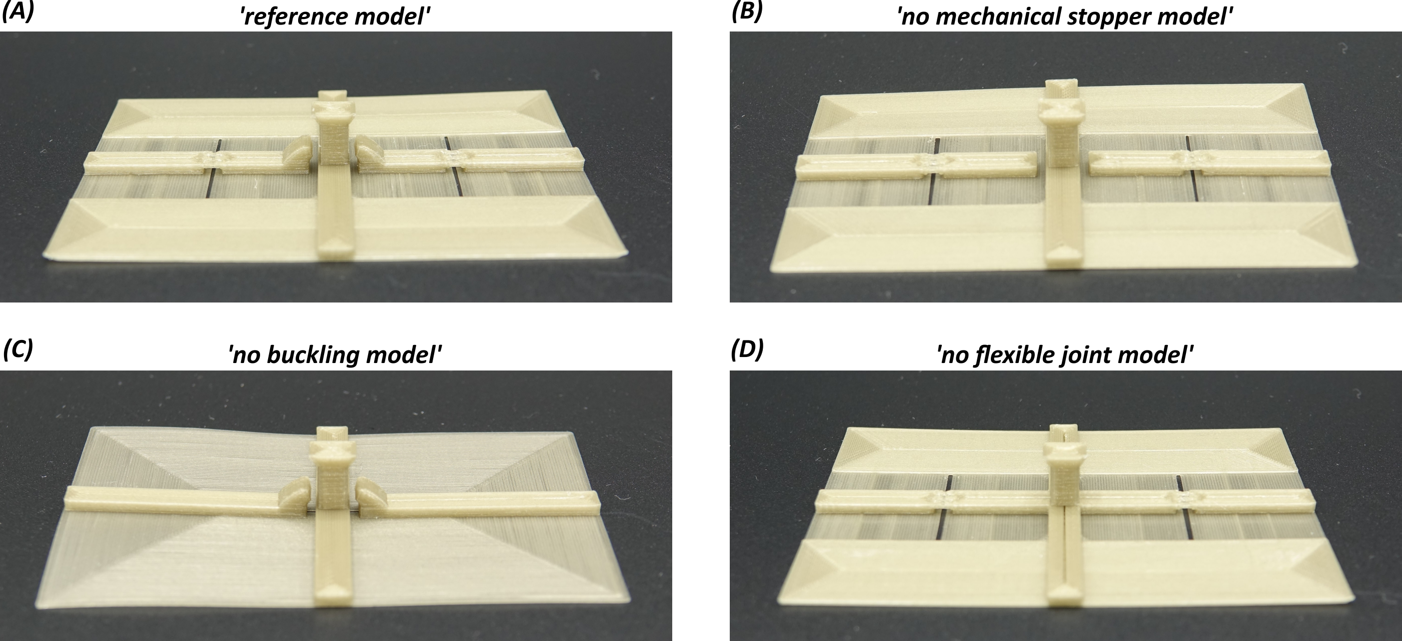
(A)
'reference model'
(B)
'no mechanical stopper model'
(C)
'no buckling model'
(D)
'no flexible joint model'

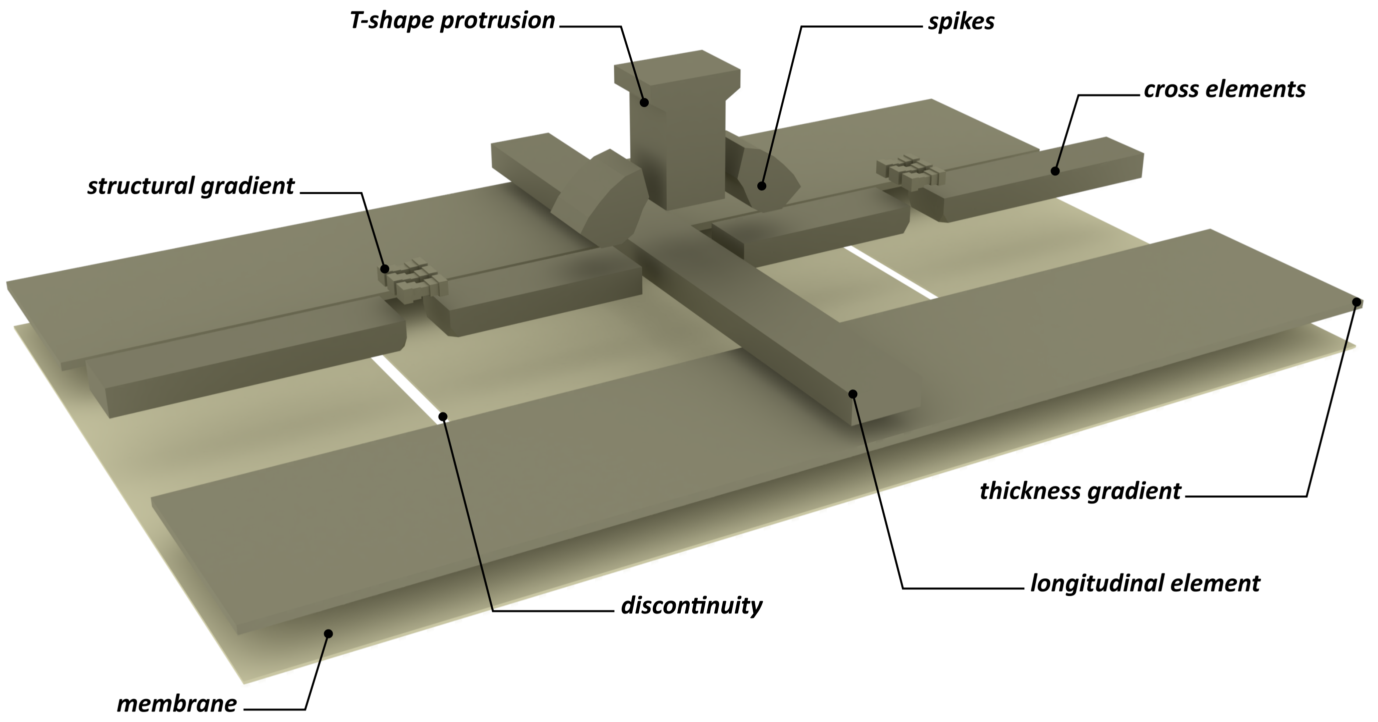
T-shape protrusion
spikes
cross elements
structural gradient
thickness gradient
longitudinal element
discontinuity
membrane
